# First Report of Safe Italian Peanut Production Regarding Aflatoxin

**DOI:** 10.3390/toxins17020090

**Published:** 2025-02-14

**Authors:** Matteo Crosta, Michele Croci, Chiara Dall’Asta, Michele Pisante, Paola Battilani

**Affiliations:** 1 Department of Sustainable Crop Production, Università Cattolica del Sacro Cuore, Via E. Parmense 84, 29122 Piacenza, Italy; matteo.crosta@unicatt.it (M.C.); michele.croci@unicatt.it (M.C.); 2 Department of Food and Drug, University of Parma, Parco Area delle Scienze 17/A, 43124 Parma, Italy; chiara.dallasta@unipr.it; 3 Department of Bioscience and Technology for Food Agriculture and Environment, University of Teramo, 64100 Teramo, Italy; mpisante@unite.it

**Keywords:** groundnut, fungi, *Aspergillus flavus*, mycotoxin, food safety

## Abstract

The growing interest in peanut production in Italy represents a significant opportunity from both an agronomic and economic standpoint. Aflatoxin B_1_ (AFB1) contamination is a major concern with imported peanuts; developing an Italian peanut supply chain can ensure a well-managed local product, with special care for food safety. This study aimed to provide a first overview of Italian peanut production, focusing on the *Aspergillus* section *Flavi* and AFB1 occurrence in the raw product. During 2022 and 2023, 18 peanut fields were sampled at complete maturity across the Italian production areas, considering three varieties: Lotos, SIS-AR_01, and IPG914. The results showed the occurrence of *Aspergillus* sec. *Flavi* in peanut pods, even though AFB1 was always absent or in traces, well below the European legal limits. These findings confirmed the quality of Italian peanut production, even though further research is requested to confirm the positive results of this first report.

## 1. Introduction

Peanut (*Arachis hypogaea* L.) is a legume native to South America that has spread worldwide over the years. In 2022 global peanut production was over 54 million tons, with China, India, Nigeria, and the USA as the leading producers [1]. During the last fifty years, the interest in this crop has grown worldwide because of the changing consumer behaviour and the search for novel protein sources for human food. This rising trend can also be found in Italy, where, since 2020, the yearly production of peanuts increased from 44.3 to 719.3 tons, and the cultivated area more than quadruplicated from 48 to 203 ha [2]. Despite this increase, the yearly Italian peanut demand, which is about 30kt, must be satisfied by imports from Egypt, Israel, the USA, and Argentina, with signalled aflatoxin contamination, mainly from African origin [3,4,5].

Aflatoxins (AFs) are toxic, carcinogenic, mutagenic, and teratogenic compounds that can cause acute or chronic aflatoxicosis [6,7,8,9]. Aflatoxin B_1_ (AFB1) is considered the most toxic compound naturally occurring in crop products, and because of its carcinogenic effect, it was classified under Group 1 (carcinogenic for humans) by the International Agency for Research on Cancer (IARC) [10]. As the presence of AFs, particularly AFB1, in food represents a threat for human health, over 100 countries around the world fixed concentration limits, EU countries included [11,12]. The limits for raw peanuts for direct human consumption or used as an ingredient in food products are 4 µg/kg of total aflatoxins (AFB1 + AFB2 + AFG1 + AFG2) and 2 µg/kg for AFB1 [11,13].

Peanuts represent a suitable substrate for the main aflatoxigenic fungi, *Aspergillus flavus* and *A. parasiticus* [14,15]. These *Aspergillus* spp. are soil-borne fungi that can be found in soil and plants [16]. These fungi can grow in temperature (T) range 12–48 °C, with optimum at 25–42 °C [16,17]. Their occurrence is mainly in tropical and sub-tropical regions between 16° and 35° of latitudes, and it is uncommon above 45° [18]. Nevertheless, climate change is causing an increase in the world areas subjected to *A.* section *Flavi* occurrence [19,20].

*Aspergillus flavus* produces AFB1 and AFB2 and cyclopiazonic acid (CPA), whereas *A. parasiticus* is capable of producing AFB1, AFB2, AFG1, and AFG2, but not CPA [21,22].

Aflatoxin production can occur in pre- and post-harvest, and it can affect many food products and crops not properly stored. Various studies were carried out to understand the optimal conditions to produce mycotoxins by fungi. Temperature and water activity (a_w_) that promotes AFs production were, respectively, 15–35 °C and a_w_ ≥ 0.85, with optimal conditions of 28–33 °C and 0.95–0.99 [23,24], even though a rapid increase in AFs synthesis was noticed with a_w_ around 0.95 in maize gain in field [25]. During the pre-harvest period, many natural environmental conditions promote AFs contamination, but high temperature and plant drought stress are considered the two main conducive factors [26,27]. They cause a reduction in plant defensive substance production, increasing the susceptibility to the attack of *A. flavus* and the potential AFs contamination [27,28]. As stated in several studies, when the geocarposphere T, in drought conditions, was between 29 °C and 31 °C, high AFs contamination levels were detected in peanut kernels [28,29,30]. However, cooler temperatures than 29–31 °C led to less AFs contamination even if the plant was in drought conditions [31]. The last 4–6 weeks of peanut-growing season, the pod maturation stage, is the most critical period for plant drought and temperature stress [29,31]. To avoid the onset of these stressful situations, irrigation is the most effective practice, particularly during the last 40–60 days of maturation [32,33,34]. To assess plant health conditions, satellite-based remote sensing techniques has been widely applied [35,36]. Sentinel-2 satellite data were exploited, identifying crop conditions and canopy water content as indicators for estimating agricultural drought in different crops, including maize, sunflowers, and peanuts [37,38,39]. Remote-sensing technologies enable near real-time monitoring and precise spatial analyses, allowing for more accurate predictions of crop stress and optimizing irrigation strategies. Integrating multispectral bands further enhances the detection of subtle variations in plant health, providing a more comprehensive view of the crop condition [40].

Considering the climate conditions and the crop chain management, Italian peanuts could be at low risk for AF contamination. However, despite the recent interest and the increase in peanut production in Italy, few studies are available in the literature [3,41,42], and none regarding the mycobiota and the AFs producing fungi occurrence in Italian peanuts. Therefore, the objectives of this work were to (i) characterize the fungal population associated with peanuts, focusing on *Aspergillus* spp., and quantify the eventual AFB1 contamination of seeds, and. (ii) study the correlation between AFB1 contamination and vegetation indices (VIs).

## 2. Results

### 2.1. Meteorological Data and Irrigation

The meteorological data considered for this study consisted of rainfall and temperature (maximum, T_max_; minimum, T_min_; mean, T_mean_; Table 1) for the whole growth period of peanut growth in field. T_max_ in 2022, as well as the T_mean_, were higher than in 2023. In 2022, only one location had a T_mean_ value below 24 °C, whereas in 2023, only two locations had a T_mean_ above this value. Rainfall in 2022 was limited, and high variability across the sampling locations was noticed, with a range from 58 to 140 mm in the five months considered. In 2023, despite the low rainfall amount in Bovolone, precipitations ranged between 98 and 165 mm in the other locations.

As for irrigation, high amounts of water were distributed in 2022, with 4–6 treatments for each location, except for Lagosanto (1 irrigation) and Cavallermaggiore (no irrigation). In 2023, irrigation was limited, except for Ostellato, where 190 mm of water were distributed during the growing season.

Table S1 reports monthly detailed meteorological data referring to peanut’s growth period (from sowing to sampling).

### 2.2. Peanut Varieties

The data presented in Table 2 show production parameters, fungal population, and AFB1 contamination for the three peanut varieties included in the study. SIS_AR_01 was distinguished by a significantly higher seed yield per pod compared to the other varieties, even though its 1000-seed weight was approximately 30% lower. In contrast, although Lotos and IPG914 had statistically comparable 1000-seed weights, seed yield was significantly higher for Lotos.

The fungal population was characterized by a high variability across varieties in terms of incidence and genera identified. *Aspergillus* section *Flavi*, the focus of this research, was isolated from all varieties, even though SIS_AR_01 showed significantly higher contamination than Lotos and IPG914. Regarding AFB1, no significant difference among varieties was noticed.

### 2.3. Production and Contamination Data for Lotos

Data about production parameters, fungal incidence, and AFB1 contamination across geographical areas and years of the Lotos variety are shown in Table 3. In 2022, peanut production reached its highest level, while in 2023, there was a significant reduction in both seed weight and shelling percentage (−9% and −5%, respectively). Areas 1, 2, and 3 showed the highest 1000 seeds weight, significantly greater than those from other areas, where the average seed weight was 26% lower. The shelling percentage was always over 70%, except for area 4, where the value was significantly lower (−9%).

High fungal incidence and genera variability occurred across years and geographic areas. The main fungi isolated belonged to *Aspergillus* spp., *Fusarium* spp., and *Penicillium* spp. However, considering *A.* sec *Flavi* and AFB1 contamination, a significant increase was observed in 2023. In particular, in 2022 the incidence of this fungus was 90% lower than in 2023, and AFB1 was not detected (see also Table S2).

### 2.4. Vegetation Indices (VIs) and AFB1

**Figure 1:**
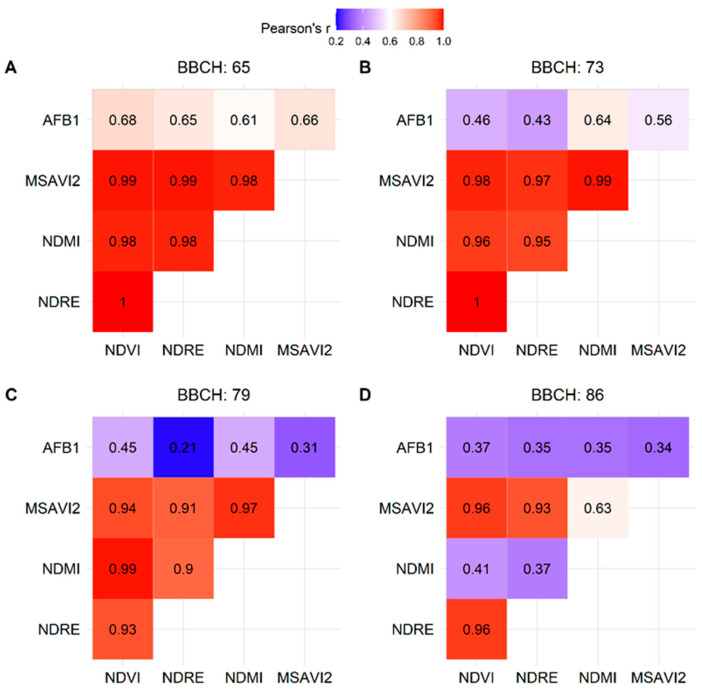
Correlation heatmaps showing Pearson’s correlation coefficients (r) between aflatoxin B1 (AFB1) contamination of peanut cultivar (cv) Lotos and vegetation indices (NDVI, NDRE, NDMI, MSAVI2) across different phenological stages of the crop, as defined by BBCH scales: (**A**) BBCH 65, (**B**) BBCH 73, (**C**) BBCH 79, and (**D**) BBCH 86.

Pearson’s correlation coefficients between AFB1 contamination and selected VIs (Table S3) are reported in Figure 1. Overall, a decreasing trend in the correlation was observed for all the VIs moving from BBCH 65 to BBCH 86. In fact, the strongest and weakest correlations were observed, respectively, during the flowering stage (BBCH 65), at the beginning of the reproductive phase, and during pod maturation (BBCH 86). NDVI generally showed a stronger relationship with AFB1 contamination compared to the other VIs, although NDMI exhibited a better correlation during pod development (BBCH 73).

## 3. Discussion and Conclusions

The growing interest in peanuts in Italy represents a significant opportunity from agronomic and local supply chain perspectives, and it contributes to the diversification of Italian crop production. Developing an Italian peanut supply chain focused on guaranteeing high-quality standards for consumers, particularly in terms of food safety, is essential for competing with the large volumes of imported products where mycotoxin contamination represents a challenge [3,5]. Within this context, the objective of the current work was to provide an overview of peanut production in Italy, with particular emphasis on *Aspergillus* sec. *Flavi* occurrence and AFB1 contamination.

To date, peanut cultivation in Italy is concentrated in the north-eastern regions, particularly in the province of Ferrara. However, the increasing national interest in this crop is demonstrated by its presence in other areas of the country, like north-west and south Italy [2,3]. Even though only one field was available in those areas for our aims, it was important to acquire preliminary data.

A relevant aspect of peanut cultivation is the choice of variety, which is constrained by the limited availability of options in Italy. In this study, the three most common varieties cultivated in Italy were considered; however, cv Lotos remains predominant in the Italian agricultural context due to the good adaptation to the Italian environment. Therefore, the disparity in varieties distribution in this study makes these results still preliminary. In recent years, considerable efforts have been done to promote the SIS_AR_01, a traditional Italian variety characterized by small seeds and intense flavour, which make it unique in the peanut market. Despite its potential, SIS_AR_01 had not yet been registered in the European Catalogue during the study (registered on February 28th, 2024, European Commission, 2024), which limited its cultivation.

Focusing on cv Lotos, the limited AFB1 contamination of mature peanuts detected highlights the high safety of Italian peanut production. AFs may occur not only before harvest but also along the whole supply chain, if not carefully managed. The application of rigorous pre-harvest crop management practices that ensure high-quality products can significantly support and simplify post-harvest operations [43], which must guarantee unsuitable conditions for fungal growth [33].

Weather conditions, particularly high temperatures and low precipitation, are the main pre-harvest factors influencing peanut yield and AF contamination [33]. Thus, it is crucial to adjust irrigation treatments to provide optimal water levels to the crop throughout the growing season [34,44,45,46]. In the current study, the two years under investigation exhibited different meteorological conditions, particularly regarding precipitation. In 2022, the weather was warmer and drier than in 2023. Despite this, frequent irrigation compensated for the low precipitation, with the total water supply (precipitation + irrigation) exceeding 300 mm in some areas. In contrast, in 2023, the water supply to the crop during the entire growing cycle was generally less than 200 mm due to reduced irrigation as a consequence of increased rainfall. This disparity resulted in a lower peanut production and an increase in *Aspergillus* sec. *Flavi* occurrence and AFB1 contamination in 2023. These findings, regarding crop yield, are consistent with those of Abou Kheira et al. [44] delivered for Egypt; they observed kernel yield reductions of approximately 36% under increasing water deficit conditions compared to full irrigation. Additionally, Sannino et al. [46] showed a significant yield increase in irrigated crops in Southern Italy.

Reduced water availability, other than influencing crop production, alters the physiological conditions of the plant, increasing its susceptibility to *Aspergillus* sec. *Flavi* infection and raising the risk of AF contamination [34,45,47]. Chalwe et al. [48] stated that a slight reduction in irrigation from 100% to 75% of the crop water requirement, from flowering to maturity, led to a significant increase in AF concentration, up to 10-fold. The impact of low water availability can be amplified when droughts occur during the early stages of the plant’s reproductive period, the peanut critical phase to *Aspergillus* sec. *Flavi* infection [49,50]. Indeed, the higher occurrence of *Aspergillus* sec. *Flavi* and AFB1 contamination observed in 2023 may be due to the timing of peanut flowering, which generally occurred in the middle of July (Table S4), a month characterised by significantly low precipitation and irrigation; in contrast, in 2022, the reproductive phase began earlier, between late June and early July, when the water supply was comparatively higher.

Early detection of water stress using VIs may play a key role in peanut pre-harvest management [38]. By monitoring parameters such as canopy greenness and leaf water status, farmers can implement timely irrigation interventions, improving water use efficiency and reducing AF contamination risk [37,38,51]. In the current work, the highest correlation between VIs and AFB1 contamination was observed at the beginning of the reproduction stage of peanuts (BBCH 65), where poor physiological condition of the plant may lead to a higher risk of contamination in peanuts at harvest. This finding agreed with the study of Boken et al. [52], where the NDVI showed a relationship (R^2^ = 0.56) with AF contamination in the early part of the reproductive phase of peanuts, even if moderate. Observing the other growth stages, a consistent decline in the correlation rate was noted for all indices except for NDMI, which slightly increased during the pod development (BBCH 73). This index is particularly sensitive to the water content of crop tissues; thus, the increase in Pearson’s rate highlighted the critical role of plant water stress during the pod formation and maturation stages in relation to AFB1 contamination [33,37].

In conclusion, this study provides evidence of the promising high food safety level of the pre-harvest Italian peanut production. The observed levels of AFB1 contamination were found to be well below the limits set out in European legislation [13], which serves to demonstrate the efficacy of the pre-harvest practices employed. Nevertheless, the difference between the 2 years considered, even if slight in terms of AF contamination, suggests that AF contamination in peanuts is not excluded, also in account of challenges posed by climatic variations, and care in crop management is a mandatory approach.

Optimal irrigation management is essential to ensure the crop’s adequate water requirements, particularly during the phase of high susceptibility to the fungus, also in years characterised by high rainfall, as evidenced by the 2023 data. The use of innovative technologies, such as early stress detection using VIs, and predictive models that provide an AFB1 contamination risk throughout the growing season [53], may be key tools to optimise irrigation practices and enhance product quality, even under adverse climatic conditions. Therefore, further investment in research, innovation, and agronomic management will be essential to harden the role of Italian peanuts as a distinctive element of national agricultural production.

## 4. Materials and Methods

### 4.1. Selection of Location and Sampling

Peanuts were sampled in 18 locations across Italy during the years 2022 and 2023 (Table 4). In 2022, ten fields were selected, seven in Ferrara and one in Modena, Cuneo, and Avellino provinces, respectively. As for Ferrara, the fields were chosen according to the peanut variety (Lotos and SIS_AR_01), the soil type (sandy, loamy, peaty), and the geolocalisation (North-West and South-East Ferrara province).

In 2023, eight locations were considered, and they were in the provinces of Ferrara (three fields), Verona (three fields), and Pordenone (two fields). Three peanut varieties were selected in 2023: Lotos, SIS_AR_01, and IPG914. Despite the local origins and the interest in the SIS_AR_01 variety, the production was still limited to a single company because of the interest in patenting the variety, which was registered in the European Common Catalogue of agricultural varieties on 28 February 2024 [54].

Given the close geographical positioning of some of the sampled locations, the fields were classified into seven distinct geographical zones.

The agronomic practices employed for the cultivation of peanuts were quite similar in the selected areas and included pre-sowing fertilization, to ensure optimal nutrient availability, and irrigation interventions during the growing cycle. Regarding sowing density, it is typically around 238,000 seeds/ha. However, with an average germination rate of approximately 72%, the resulting investment was about 171 thousand plants/ha.

Five sampling points were defined in each field at the complete maturity stage [53], immediately after digging, following the field’s diagonals. A sample of approximately 200 g of peanuts was collected in each sampling point, with five replicates for each field (10 fields in 2022 and 8 fields in 2023). The collected samples were managed to keep only not damaged, clean (without soil) pods.

### 4.2. Meteorological Data and Irrigation

Meteorological data were collected through the Meteoblue web platform, which provides georeferenced data for the fields of interest. This virtual service is based on the interpolation of data from physical weather stations, by which 4 × 4 km cells are obtained. Meteorological data for the whole growing period of peanuts (Table 4) and water applied to the selected fields through irrigation were supplied by the company DIAGRAM Group. The data were collected from the sowing to the complete maturity (sampling time) of peanuts.

### 4.3. Fungal Population Analysis

#### 4.3.1. Sample Preparation

Each sample was dried at 40 °C for four hours, using a stove (TCN 200 Plus, Argolab, Carpi, MO, Italy) to improve the shelf life until the analysis. Subsequently, 50 pods from each replicate were weighted (Sauter RC 4021, Basel, Switzerland) and shelled to count and weight the seeds. The 1000 seed weight and the shelling percentage ([55] modified) for each of the samples were calculated using the respective equations:

(1) $$1000\;seed\;weight=\frac{seed\;sample\;weight}{number\;of\;seeds}*1000$$

(2) $$shelling\;percentage=\frac{seeds\;weight}{pods\;weight}*100$$

subsequently, the seeds were ground using a blender (Blendforce Glass Blender, Moulinex, Écully, France) and the resulting powder was used in the next analysis.

#### 4.3.2. Fungal Identification

The serial dilution method was carried out to assess the fungal population of peanuts. One g of each ground sample was added to 9 mL peptone physiological solution 1% (PPS) in sterile tubes and stirred using a vortex. Then, starting from the homogenized samples, four serial dilutions were made in PPS (10^−2^ to 10^−5^), and 1 mL from each diluted solution was spread-plated on Dichloran-Rose Bengal Chloramphenicol Agar plates (DRBC; Biolife, Monza, Italy) in triplicate. The plates were incubated at 25 °C for 6 days and developed colonies were counted, classified at genus level based on colony morphology, and reported as colony-forming units per gram (CFU/g). To better identify the genera of fungi, representative colonies were transferred into potato dextrose agar (PDA; Biolife, Monza, Italy) plates and then incubated at 25 °C for seven days. After incubation, the identification was carried out by the macro and microscopic observation of the colonies according to Pitt and Hocking [56].

### 4.4. Aflatoxin Analysis

#### 4.4.1. Reagents and Chemicals

Aflatoxin mixture (AFB1, AFB2, AFG1, and AFG2) analytical standard was purchased from Sigma Aldrich (Stenheim, Germany). Ultrapure water, acetonitrile (MeCN), and formic acid (FA) were obtained from Scharlab Italia Srl (Milan, Italy), sodium chloride (NaCl) and anhydrous magnesium sulfate (MgSO_4_), Z-sep+ and C18 sorbent, form VWR international, Milan, Italy. The mobile phase A was a 0.2% acetic acid with 5 mM ammonium acetate solution. Mobile phase B consisted of methanol (MeOH), water, and acetic acid (97:2:1 *v*/*v*). MeOH was used as phase C and ultrapure HPLC-graded water as phase D.

#### 4.4.2. Aflatoxins Extraction

The extraction procedure was based on a routinary method in used in our laboratory, based on Sartori et al. [57] with slight modifications.

Briefly, one gram of the homogenised ground peanuts from three replicates of each sample were weighed and added into a 50 mL polypropylene centrifuge tube together with a spike concentration of 10 ppb AFs standard mixture and 5 mL of water. To find the right approach a distinction between spiking in the beginning and spiking at the end was made. Subsequently, the tubes were put into a mechanical shaker for 10 min at 200 strokes/min. In this approach, the QuEChERS method was followed. Amounts of 5 mL of MeCN with 5% FA solution, 2 g of MgSO_4_, and 0.5 g of NaCl were added. Immediately after, the tubes were shaken by hand and vortexed for 30 s and, to induce phase separation, the samples were centrifuged for 5 min at 3700 rpm. At this point, 1.5 mL of the resulting supernatant was taken and transferred into a tube containing 50 mg C18 and 50 mg Z-sep+. The tubes were centrifuged for 3 min at 1750 rpm, and 1.25 mL of the supernatant, from each sample, were added into clean vials. The vials were dried under a gentle nitrogen flow. Dried samples were reconstituted with 250 µL of mobile phase B vortexed until there were no more residues left and injected into the LC-MS/MS system.

#### 4.4.3. LC-MS/MS Analysis

The analysis was conducted using a Thermo Scientific Dionex Ultimate 3000 instrument coupled with a triple quadrupole mass spectrometer (TSQ Vantage, Thermo Fisher Scientific Inc., San Jose, CA, USA). The separation was obtained with a Kinetex column, 2.6 µm Evo C18 (Phenomenex, Milan, Italy), 100 × 2.1 mm, heated to 40 °C. Two µL of each sample was injected with a flow rate set up to 0.4 mL/min. Gradient elution was achieved by using mobile phases A and B. The total run time lasted 18 min and the gradient was set as follows: 2% B for 1 min, and then B reached 90% in 8 min; a constant gradient was kept for 3 min until a rapid decrease in B to the initial concentration after 13 min from start; finally, this gradient was maintained for the last 5 min before following injection. MS analysis was performed in positive ionization mode using SRM as a monitoring method. The following quantifier transitions were evaluated: AFB1 m/z 313.1 > 241.2 (CE 42eV), 313.1 > 270.1 (CE 28eV), 313.1 > 285.1 (CE 25eV); AFB2 *m*/*z* 315.2 > 259 (CE 30eV), 315.2 > 287 (CE 25eV); AFG1 *m*/*z* 329 > 243 (CE 25eV), AFG1 329 > 311 (CE 20eV); AFG2 *m*/*z* 331.3 > 245.3 (CE 25eV), AFG2 331.3 > 270 (CE 30eV), AFG2 331.3 > 285 (CE 30eV), AFG2 331.3 > 313.3 (CE 30eV). Regarding the MS parameters for the analysis, the spray voltage was set at 3500 V, while the temperature of the capillary and of the vaporizer were set at 270 °C and 200 °C, respectively. The sheath gas flow was set at 50 units, and the auxiliary gas flow at 5 units. Calibration curves were set up using external standards (range 1 µg/kg to 500 µg/kg). Data acquisition and processing was performed by Thermo Xcalibur 2.2 software (Thermo Fisher Scientific, Waltham, MA, USA).

A matrix-matched calibration was performed in the range 0.5–100 µg/Kg, using a blank peanut extract spiked at 5 concentration levels. Each concentration level was prepared and analysed in triplicate. Linearity was satisfactory in the selected range, with calibration curves presenting r2 > 0.995. LOQ was set at the lowest calibration point (0.5 µg/kg) for all mycotoxins. LOD was determined as signal-to-noise ration and about 0.1 µg/Kg for all mycotoxins. Recovery was calculated at the LOQ and at 10 µg/Kg, presenting values in the range 95–110% for AFB1, AFB2, AFG1 and AFG2. The analysis was stable over time, with a retention time error ≤ 1.1% for all the analytes.

### 4.5. Satellite Imagery

Spectral data for Lotos fields affected by *Aspergillus flavus* infection were analysed using the Google Earth Engine (GEE) cloud computing platform [58]. Copernicus Sentinel-2 A and B satellite images were processed, with all spectral bands resampled to a spatial resolution of 10 m, and a series of VIs were then calculated. The analysis focused on images acquired between May 1 and October 31, covering the whole peanut growing period, limiting the selection to scenes with less than 65% cloud cover.

The calculated VIs included the following: Normalized Difference Moisture Index (NDMI; [59]), Normalized Difference Red-Edge Index (NDRE; [60]), Normalized Difference Vegetation Index (NDVI; [61]), and Modified Soil-Adjusted Vegetation Index 2 (MSAVI2; [62]). After downloading the satellite images, the AF values measured at each sampling point were matched with the VI values (Table S3) of the corresponding satellite pixels calculated for four peanut growth stages selected during the reproductive period (Table 5).

### 4.6. Statistical Analysis

Statistical data analysis was carried out by applying the analysis of variance (ANOVA) and Tukey’s HSD test (α = 0.05) to determine the presence of significant differences between the samples’ means. All data about the fungal population of the samples were transformed by the natural logarithm (y = ln(x + 1)) to homogenize the variance, and yield percentages were arcsin-transformed (y = arcsin (√x)) before applying the ANOVA test. These analyses were made by the statistical package IBM SPSS Statistics 27 (IBM Corp., Armonk, NY, USA). Two distinct ANOVA analyses were performed considering the imbalance of peanut fields and varieties across the years. The first ANOVA was conducted to examine the influence of varieties on fungal population and production parameters. The subsequent analysis was limited to Lotos variety to undertake a more comprehensive investigation on the impact of geographical regions and years on the studied variables.

A Pearson correlation analysis [64] was carried out to define the relationship between AFB1 concentration and VI values.

## Figures and Tables

**Table 1:** Summary of the mean meteorological data (T_max_, T_min_, T_mean_, and precipitations) and irrigation data collected during the 2-year study (2022 to 2023) in the sampled locations during the whole growth period of peanuts (from sowing to sampling).

Area	Province	Long	Lat	Year	T_max_ (°C)	T_min_ (°C)	T_mean_ (°C)	Precipitations (mm)	Irrigation (n)	Irrigation (mm)
1	Ferrara	11.32894	44.75973	2022	32.12	16.53	24.51	123.90	5	189
	Modena	11.28777	44.85639	2022	32.66	16.94	25.01	115.70	6	214
	Ferrara	11.48193	44.88769	2023	31.22	17.45	24.54	143.50	1	40
2	Ferrara	12.18881	44.78862	2022	29.75	18.64	24.29	63.60	1	48
	Ferrara	12.07147	44.91150	2022	31.69	18.37	24.89	67.00	4	155
	Ferrara	12.06913	44.91158	2022	31.25	17.99	24.50	93.70	4	204
	Ferrara	12.05803	44.88688	2023	28.77	17.98	23.33	165.71	NA *	NA
3	Ferrara	12.04619	44.69533	2022	31.90	18.31	25.02	60.10	5	228
	Ferrara	12.11603	44.72886	2022	31.02	18.66	24.80	58.10	5	234
	Ferrara	11.92636	44.62356	2022	31.84	17.72	24.75	63.50	5	175
	Ferrara	11.89168	44.74335	2023	31.18	17.89	24.59	98.90	5	190
4	Verona	11.10991	45.27143	2023	29.81	18.25	23.89	55.40	5	125
	Verona	11.10933	45.27024	2023	29.77	18.43	23.97	55.40	5	125
	Verona	11.11122	45.26857	2023	29.77	18.43	23.97	55.40	5	125
5	Pordenone	12.88952	45.86698	2023	30.31	17.35	23.84	149.80	0	0
	Pordenone	12.91461	45.87008	2023	30.33	17.62	24.00	148.40	1	30
6	Cuneo	7.68155	44.71550	2022	30.24	16.75	23.60	140.10	0	0
7	Avellino	14.77357	40.82467	2022	NA	NA	NA	NA	NA	NA

* NA: Not Available.

**Table 2:** Production parameters (weight of 50 pods and seeds, number of seeds contained in 50 pods, shelling percentage, mean number of seeds per pod and 1000 seeds weight), fungal incidences, and AFB1 contamination, of three peanut varieties (Lotos, IPG914, and SIS_AR_01). The results of the analysis of variance (ANOVA) and Tukey’s HSD post hoc test (α = 0.05) are reported.

**Factor**	**Pods Weight (g)**	**Seeds Weight (g)**	**Number of Seeds (n)**	**Shelling Percentage % ^1^**	**Mean Seeds per Pod (n)**	**1000 Seeds Weight (g) ^2^**
Variety	**	**	**	**NS**	**	**
Lotos	143.72 a	102.79 a	109.43 b	71.48	2.2 b	937.18 a
IPG914	111.10 b	78.61 b	93.50 c	70.99	1.9 c	840.31 a
SIS_AR_01	136.61 a	98.90 a	158.30 a	72.18	3.2 a	625.23 b
**Factor**	***Aspergillus*** **sec. *Flavi*** **(CFU/g)**	***Aspergillus*** **sec. *Nigri*** **(CFU/g)**	***Fusarium*** **spp. (CFU/g)**	***Penicillium*** **spp. (CFU/g)**	**Total fungi (CFU/g)**	**AFB1 (µg/kg)**
Variety	**	*	**NS**	**	**NS**	**NS**
Lotos	2.11 × 10^1^ b	1.10 × 10^4^	3.73 × 10^3^	4.54 × 10^4^ ab	1.01 × 10^5^	0.18
IPG914	4.00 × 10^1^ b	2.17 × 10^2^	1.77 × 10^2^	2.40 × 10^2^ b	1.56 × 10^3^	0.00
SIS_AR_01	6.44 × 10^2^ a	4.63 × 10^3^	0.00 × 10^0^	2.09 × 10^4^ a	2.73 × 10^4^	0.39

* (*p* < 0.05); ** (*p* < 0.01); “NS” Not Significant; different letters indicate significant differences within the same year, according to Tukey’s HSD test. ^1^ The percentage was calculated by the equation y = (seeds weight/pods weight) × 100. ^2^ 1000 seeds weight was calculated by the equation y = (seeds weight/number of seeds) × 1000.

**Table 3:** Production parameters (weight of 50 pods and seeds, number of seeds contained in 50 pods, shelling percentage, mean number of seeds per pod and 1000 seeds weight), fungal incidences, and AFB1 contamination of peanut Lotos variety collected in seven geographical areas across 2022 and 2023. Results of the analysis of variance (ANOVA) and Tukey’s HSD post hoc test (α = 0.05).

**Factors**	**Pods Weight (g)**	**Seeds Weight (g)**	**Number of Seeds (n)**	**^2^ Shelling Percentage %**	**Mean Seeds per Pod (n)**	**^3^ 1000 Seeds Weight (g)**
Geographical area ^1^	**	**	**	**	**	**
1	162.25 a	115.95 a	112.73 a	71.52 a	2.25 a	1028.30 a
2	155.28 a	113.27 a	111.10 a	72.91 a	2.22 a	1021.13 a
3	150.40 ab	110.08 a	110.45 a	73.27 a	2.21 a	998.09 a
4	135.71 bc	89.30 bc	109.40 ab	65.85 b	2.19 ab	816.33 b
5	126.84 cd	91.38 b	111.40 a	72.03 a	2.23 a	821.22 b
6	106.70 e	76.58 c	100.00 b	71.74 a	2.00 b	766.39 b
7	108.20 de	77.76 bc	99.60 b	71.85 a	1.99 b	780.72 b
Year	**NS**	**	**NS**	**	**NS**	**
2022	145.79	106.24 a	109.0	72.87 a	2.18	971.24 a
2023	140.00	96.58 b	110.3	68.99 b	2.21	875.87 b
**Factors**	***Aspergillus*** **sec. *Flavi*** **(CFU/g)**	***Aspergillus*** **sec. *Nigri*** **(CFU/g)**	***Fusarium*** **spp. (CFU/g)**	***Penicillium*** **spp. (CFU/g)**	**Total fungi (CFU/g)**	**AFB1 (µg/kg)**
Geographical area	**NS**	**	*	**NS**	*	**NS**
1	9.52 × 10^0^	3.15 × 10^2^ bc	1.45 × 10^4^	7.63 × 10^4^	2.07 × 10^5^ a	0.10
2	0.00 × 10^0^	6.93 × 10^4^ b	7.00 × 10^1^	3.29 × 10^4^	1.58 × 10^5^ a	0.00
3	1.50 × 10^1^	3.17 × 10^2^ bc	8.62 × 10^2^	8.52 × 10^4^	8.68 × 10^4^ ab	0.04
4	3.20 × 10^1^	2.14 × 10^2^ b	1.10 × 10^1^	9.28 × 10^1^	6.79 × 10^2^ b	0.66
5	1.20 × 10^2^	9.90 × 10^3^ a	3.53 × 10^2^	4.71 × 10^3^	1.54 × 10^4^ ab	0.76
6	2.00 × 10^1^	0.00 × 10^0^ c	5.34 × 10^3^	8.20 × 10^2^	6.61 × 10^3^ ab	0.00
7	0.00 × 10^0^	0.00 × 10^0^ c	1.73 × 10^3^	7.23 × 10^2^	1.30 × 10^5^ ab	0.00
Year	*	**	**NS**	**NS**	*	*
2022	4.92 × 10^0^ b	1.59 × 10^4^ a	5.80 × 10^3^	7.01 × 10^4^	1.56 × 10^5^ a	0.00 b
2023	4.95 × 10^1^ a	2.32 × 10^3^ b	1.03 × 10^2^	1.99 × 10^3^	4.98 × 10^3^ b	0.50 a

^1^ The locations of each geographical area are reported in Table 4. * (*p* < 0.05); ** (*p* < 0.01); “NS” Not Significant; different letters indicate significant differences within the same year, according to Tukey’s HSD test. ^2^ The percentage was calculated by the equation y = (seeds weight/pods weight) × 100. ^3^ 1000 seeds weight was calculated by the equation y = (seeds weight/number of seeds) × 1000.

**Table 4:** Peanut fields selected for the study in 2022 and 2023 in different locations, attributed to 7 geographical areas. Coordinates, cultivation year, peanut variety, previous crop, sowing and sampling time were reported.

Area	Province	Long	Lat	Year	Peanut Variety	Previous Crop	Sowing Time	Sampling Time
1	Ferrara	11.328936	44.75973	2022	Lotos	Maize	10/05	04/10
1	Modena	11.287771	44.856394	2022	Lotos	Maize	19/05	29/09
1	Ferrara	11.481934	44.887692	2023	Lotos	Wheat	30/05	29/09
2	Ferrara	12.188809	44.788623	2022	Lotos	Chard seed	10/05	07/09
2	Ferrara	12.071466	44.911498	2022	Lotos	Wheat	09/05	16/09
2	Ferrara	12.069126	44.911584	2022	SIS_AR_01	Wheat	09/05	22/09
2	Ferrara	12.058032	44.886884	2023	SIS_AR_01	NA *	11/05	21/09
3	Ferrara	12.046193	44.695327	2022	Lotos	Maize	10/05	13/09
3	Ferrara	12.116027	44.728864	2022	Lotos	Maize	10/05	10/09
3	Ferrara	11.926363	44.623563	2022	Lotos	Ryegrass	11/05	22/09
3	Ferrara	11.891678	44.743345	2023	Lotos	Soybean	27/05	21/09
4	Verona	11.109907	45.271434	2023	IPG914	Mixed crops	15/06	11/10
4	Verona	11.109327	45.270235	2023	Lotos	Mixed crops	14/06	03/10
4	Verona	11.111219	45.26857	2023	Lotos	Mixed crops	14/06	03/10
5	Pordenone	12.889521	45.866977	2023	IPG914	Soybean	29/06	11/10
5	Pordenone	12.914613	45.870081	2023	Lotos	Soybean	27/06	03/10
6	Cuneo	7.681548	44.7155	2022	Lotos	Fallow field	07/05	28/09
7	Avellino	14.77357	40.824669	2022	Lotos	NA	NA	NA

* NA: Not Available.

**Table 5:** Peanut phenological stags selected for calculating vegetation indices and description according to Meier [63].

BBCH	Phase Description
65	Flowering
73	Pods development and pod filling
79	Seeds fill the inner space of the pods, which have reached their full size
86	60% of fully developed pods are ripe

## Data Availability

The original contributions presented in this study are included in this article and supplementary material. Further inquiries can be directed to the corresponding author.

## Supplementary Materials

The following supporting information can be downloaded at: https://www.mdpi.com/article/10.3390/toxins17020090/s1, Table S1: summary of the monthly mean meteorological data (Tmax, Tmin, Tmean, and precipitations), irrigation data collected during the 2-year study (2022 to 2023), and the water supply in the sampled locations during the whole growth period of peanuts (from sowing to sampling, Table 4); Table S2: Summary of aflatoxin (B1-AFB1, B2-AFB2, G1-AFG1, G2-AFG2) analysis results for three peanut varieties collected from seven geographic areas in 2022 and 2023. Table S3: summary of vegetation indices’ values calculated for Lotos field showing *Aspergillus*. sec. *Flavi* infection at selected BBCH stages (Table 5); Table S4: summary of selected main Lotos growth stages and the corresponding date.
